# MR spectroscopy of the cerebellum in gluten ataxia and gluten encephalopathy

**DOI:** 10.1007/s00415-026-14200-6

**Published:** 2026-10-13

**Authors:** M. Hadjivassiliou, RA. Grunewald, I. Croal, N. Hoggard

**Affiliations:** 1 Academic Department of Neurosciences, Sheffield Teaching Hospitals NHS Trust, Sheffield, UK; 2 School of Medicine and Population Health, University of Sheffield, Sheffield, UK

**Keywords:** Gluten sensitivity, Gluten ataxia, Gluten encephalopathy, MR spectroscopy of the cerebellum

## Abstract

**Objective:**

To identify any MR spectroscopy differences amongst patients with gluten ataxia and patients with gluten encephalopathy and interpret what this tells us about cerebellar functionality.

**Methods:**

Retrospective review of MR spectroscopy baseline data from 40 patients with gluten ataxia (GA), 40 patients with gluten encephalopathy (GE) and a group of 55 healthy controls.

**Results:**

The mean age at development of ataxia in the GA group was 50 years. In the GE group, the mean age at development of neurological symptoms was 37 years. The mean age of the healthy control group at the time of scanning was 41 years. Patients with GA showed highly significant reduction in the NAA/Cr (N-acetyl-aspartate/creatine) ratio from the vermis NAA/Cr 0.85 vs 0.99 in healthy controls (*p* = 0.0000105, Welch’s *t*-test). Within this same group, the right hemisphere NAA/Cr area ratio was 0.969 vs 1.01 in healthy controls (*p* = 0.058). By contrast, patients with GE showed significant reduction of NAA/Cr area ratio in the right hemisphere 0.926 vs 1.01 in healthy controls (*p* = 0.00053, Welch’s *t*-test) but no difference from control subjects when comparing vermis NAA/Cr.

**Conclusions:**

MR spectroscopy of the cerebellum demonstrates dysfunction in the vermis in patients with GA, whereas patients with GE have abnormal spectroscopy in the right cerebellar hemisphere in the absence of ataxia. The right cerebellar hemisphere may be implicated in cerebellar cognitive affective syndrome.

## Introduction

The commonest neurological manifestations of the umbrella term gluten sensitivity (which includes coeliac disease—CD) in order of frequency are ataxia (gluten ataxia—GA), peripheral neuropathy (gluten neuropathy—GN) and gluten encephalopathy (GE) [1]. GE encompasses a combination of symptoms including cognitive slowness, episodic headaches (usually migraine-like), unexplained sensory symptoms and sometimes white matter, often referred to as “vascular” changes, on MRI brain imaging [2]. Patients with GE often complain of “foggy brain”, slow mentation and inability to think straight. These symptoms can be troublesome and often interfere with the ability to work and perform other everyday activities. Characteristically, patients often report dramatic response of these symptoms following a strict gluten-free diet (GFD) with often complete elimination of the headaches, improved cognition and clarity of thought and resolution of the sensory symptoms [3].

The cerebellar vermis is primarily involved in GA. This is based on imaging work including MR spectroscopy of the cerebellum showing preferential involvement of the vermis [4]. This finding is in keeping with the clinical picture of primarily gait ataxia which is the commonest feature of GA. Vermian involvement is also characteristic of other immune-mediated ataxias [5].

Whilst imaging findings in GE may demonstrate some white matter changes, often referred to as “vascular”, only a small proportion of GE patients have such abnormalities. The pathophysiology of GE therefore remains unresolved. The cerebellum is almost universally involved in the context of gluten sensitivity and CD, and has been increasingly recognised as an important region for cognitive as well as motor function. Arguably, most relevant is the characterisation of cerebellar cognitive affective syndrome (CCAS): the specific constellation of cognitive and social/emotional dysfunction associated with cerebellar injury [6]. This psychological phenotype shares only some overlap with broader pictures of impairment such as that due to dementia. The primary assessment for CCAS accordingly includes a number of sub-tasks found in common batteries like the Montreal Cognitive Assessment, such as phonemic fluency and delayed recall, but also deviates by emphasising tasks of category switching, inhibition and affect, in place of e.g. serial subtraction and naming.

Whilst cerebellar dysfunction could therefore contribute to cognitive deficit in both GA and GE, the different presentation of these two patient groups may indicate unique pathophysiology within that region. We therefore reviewed any MR spectroscopic differences between patients with GA and those with GE.

## Methods

This retrospective data analysis was given ethical approval via the East Midlands REC (IRAS 327729). Informed consent was waived because the study was retrospective and reported on outcomes based on standard clinical care.

Patients with neurological manifestations related to gluten sensitivity routinely undergo MR spectroscopy of the cerebellar vermis and right hemisphere at baseline (before commencing a gluten-free diet) to provide information on cerebellar N-acetyl-aspartate/creatine (NAA/Cr) ratio in each region [4]. All patients referred to in this work were seen at the dedicated gluten sensitivity/neurology clinic, Sheffield Teaching Hospitals NHS Trust. We selected 40 consecutive patients from each group (40 with GA only and 40 with GE only—see below) for the purpose of this comparison. We compared the findings in the two groups with normative data based on 55 healthy adults (using Welch’s *t*-test) collected by our group previously [7]. Planned comparisons were 1: spectroscopy values from the right cerebellar hemisphere within the groups, gluten encephalopathy and gluten ataxia, and 2: spectroscopy values from the cerebellar vermis in the two groups by unpaired *t*-test. A *P* value of less than 0.05 was considered significant.

Patients were diagnosed as having GA or GE based on the presence of circulating antibodies (one or more of the following: tissue transglutaminase 2, endomysium, antigliadin IgG and/or IgA, TG6 antibodies IgG and/or IgA) and in the absence of an alternative aetiology for their neurological presentation. Patients with GA presented with gait instability, and sometimes with other features of ataxia (limb ataxia, nystagmus, slurred speech). Patients with GE complained of “foggy brain”, slow mentation, intermittent but sometimes intractable headaches, cognitive difficulties and sometimes unexplained sensory symptoms. Inevitably some patients complained of a mixture of both ataxia and encephalopathy and sometimes peripheral neuropathy. If patients had a combination of both GA and GE, they were excluded from this analysis as were patients with gluten neuropathy (neurophysiological evidence of a peripheral neuropathy).

We compared MR spectroscopy NAA/Cr ratio obtained for the vermis and right hemisphere in each group. Details of the MR spectroscopy technique can be found in previous publications from our group [4]. Both groups underwent the same scanning sequences and were scanned on the same scan. All scans were reported by the same neuroradiologist.

## Results

**Fig. 1:**
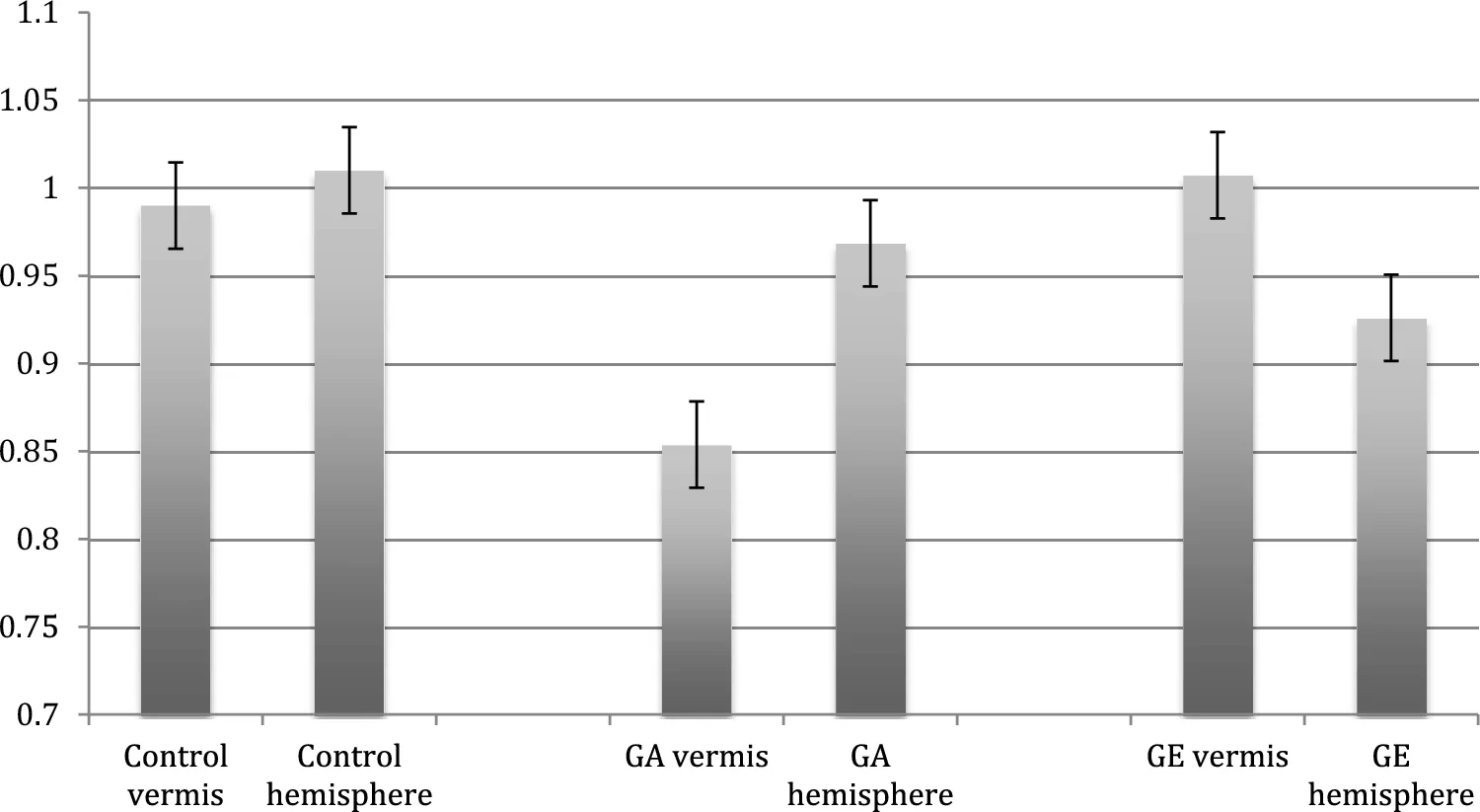
MR spectroscopy of the cerebellum (vermis and right hemisphere) in healthy controls, patients with gluten ataxia (GA) and patients with gluten encephalopathy (GE). The vertical axis refers to the NAA/Cr ratio (N-acetyl-aspartate/creatine). In GA, it is the vermis that is primarily affected (in keeping with the clinical presence of gait ataxia), whilst in GE it is the right hemisphere that is affected, manifesting primarily with cognitive dysfunction, headaches and sometimes unexplained sensory symptoms

**Fig. 2:**
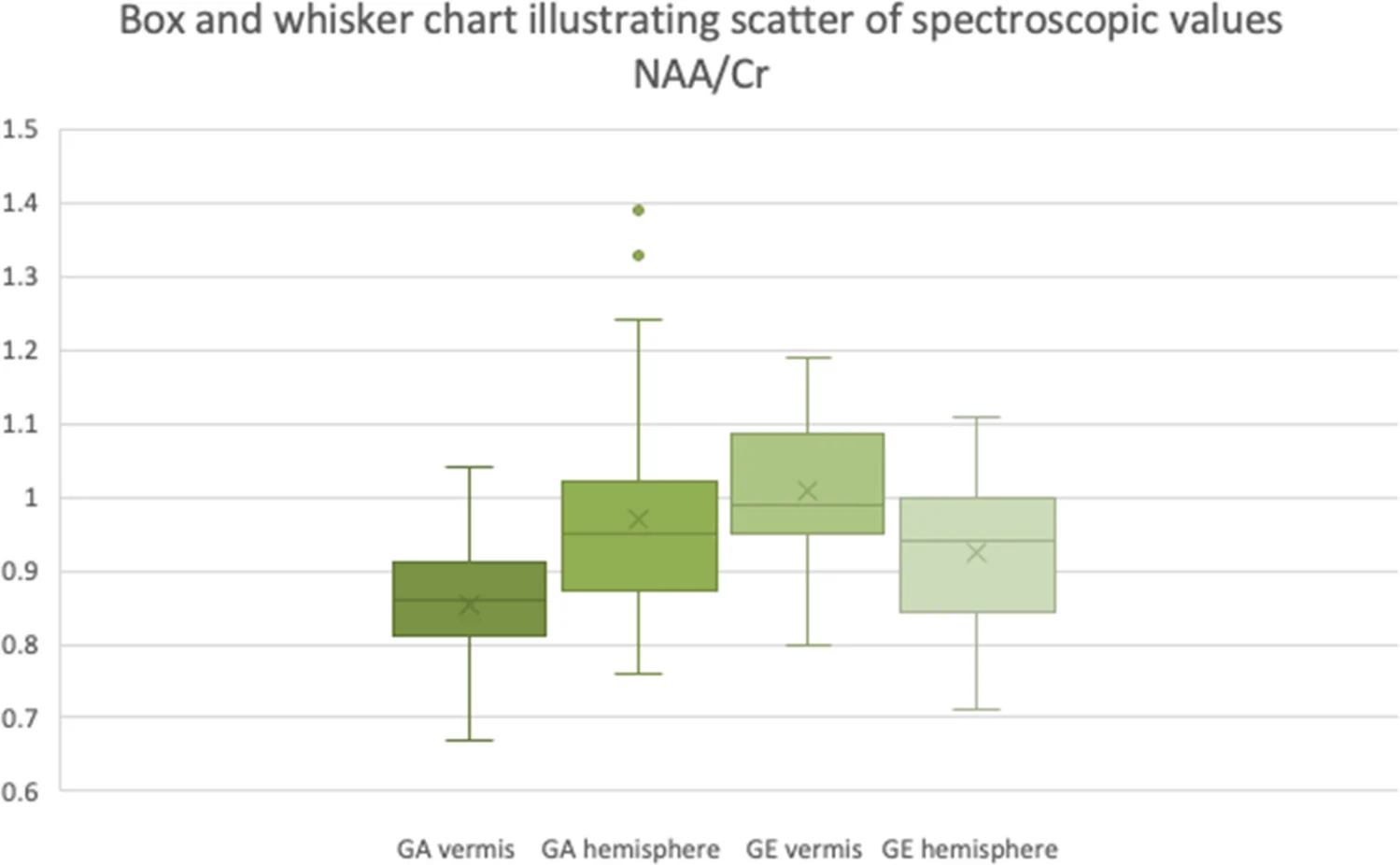
Spectroscopic values (N-acetyl aspartate/creatine-NAA/Cr) in the vermis and right hemisphere of the two groups (box and whisker chart). GA—gluten ataxia, GE—gluten encephalopathy

The mean age at development of neurological symptoms in the GA group was 50 years and the mean duration of their symptoms at the time of the scanning was 11 years. In the GE group, the mean age at development of symptoms was 37 years and the mean duration of their symptoms was 11 years. The mean age of the healthy control group at the time of scanning was 41 years. Patients with GA showed highly significant reduction in the NAA/Cr area ratio from the vermis NAA/Cr 0.85 vs 0.99 in healthy controls (*p* = 0.0000105, Welch’s *t*-test). Within this same group, the right hemisphere NAA/Cr area ratio was 0.969 vs 1.01 in healthy controls (*p* = 0.058), a difference which was not significant. By contrast, patients with GE showed significant reduction of NAA/Cr area ratio in the right hemisphere 0.926 vs 1.01 in healthy controls (*p* = 0.00053, Welch’s *t*-test), but no difference from control subjects when comparing vermis NAA/Cr. The differences in MR spectroscopy between the three groups is illustrated in Fig. 1. A direct comparison of the NAA/Cr values in the GA and GE groups showed: cerebellar vermis 0.85375 in GA vs 1.00725 in GE *p* < 0.0001 and right cerebellar hemisphere 0.9685 in GA vs 0.926 in GE, *P* = 0.12 (unpaired *t*-test). The distribution of spectroscopic values in the patient groups is illustrated in Fig. 2 by a box and whisker chart.

## Discussion

In this report, we demonstrate that patients with GE are more likely to have significant dysfunction of the right cerebellar hemisphere rather than the vermis as demonstrated with the use of MR spectroscopy of the cerebellum (low NAA/Cr area ratio). By contrast, the opposite was observed in patients with GA (the vermis is involved much more than the hemisphere). We cannot comment on the left cerebellar hemisphere because we do not routinely measure spectroscopy from there. There is existing evidence, however, that lesions of the right cerebellar hemisphere result in greater cognitive deficits than lesions of the left hemisphere [8].

NAA reflects healthy metabolic activity whilst creatine is a stable metabolite. Reduction in NAA reflects the presence of cerebellar dysfunction. As creatine does not change in disease states, the ratio of NAA/Cr provides a numerical ratio that reflects cerebellar function/dysfunction [9]. It can also be used as a monitoring tool. In fact, we have already demonstrated improvement in the NAA/Cr ratio in patients with GA after the introduction of GFD [4].

The difference in age between the group with GA and that with GE concurs with our experience that patients with GE present at a younger age when compared to patients with GA. This age difference is unlikely to account for the differences on MR spectroscopy given that both groups had significant abnormalities when compared to healthy controls and the mean age of the healthy controls was higher than the GE group.

The important role of the cerebellum in cognition has been a more recent discovery primarily thanks to the pioneering work of Schmahmann and his colleagues [10]. The term cerebellar cognitive affective syndrome (Schmahmann syndrome) now refers to a constellation of deficits in the cognitive domains of executive function, spatial cognition, language and affect.

Patients with GE characteristically complain of “foggy brain”, slow mentation, inability to think straight, often with slow speed of processing, and frequently with intermittent migraine-like headaches. These symptoms can be severe enough to interfere with ability to work and other daily activities. Characteristically patients often report striking response of these symptoms following a strict gluten-free diet (GFD) with often complete elimination of the headaches (usually within weeks), improved cognition and clarity of thought and resolution of the sensory symptoms (between 6 months and a year after the introduction of the GFD) [3].

A recent study from our group demonstrated the high prevalence of cerebellar cognitive affective syndrome using the cognitive scale (CCAS-S) in patients with neurological manifestations of gluten sensitivity (including GA and GE) attending a dedicated gluten sensitivity/neurology clinic: definite CCAS was found in 39.2%, probable in 26.4%, possible in 18.4% with only 16% not having any such cognitive issues [11]. Given that the majority of these patients were already on a GFD, the scale of the problem is likely to be much more, given that GFD improves the spectroscopic findings in parallel to the clinical improvement. More importantly, the same study demonstrated that ongoing serological positivity for IgA TG6 antibodies (a more sensitive marker for gluten sensitivity-related neurological dysfunction) was associated with more regional brain atrophy. Furthermore, this group of patients reported greater depression, symptom severity and poorer physical functioning.

Vermian involvement in the context of GA is in keeping with the typical phenotype of primarily gait ataxia in this group of patients. In fact, such involvement is typical in many other immune-mediated ataxias [12]. By contrast, patients with GE in isolation do not complain of balance issues or have much evidence of cerebellar ataxia on clinical examination. In fact as part of the analysis, here we had excluded any patients who had a mixed picture of both ataxia and encephalopathy. Yet, MR spectroscopy demonstrates significant dysfunction in the right cerebellar hemisphere in this group of patients with GE when compared to healthy individuals. It is important to clarify that GE does not equate with CCAS. It appears, however, that in the context of gluten sensitivity, involvement of the right cerebellar hemisphere may manifest primarily, if not exclusively, with cognitive limitations rather than ataxia. The reason why some patients present exclusively with GE whilst others present exclusively with GA remains unclear, but the universal involvement of the cerebellum in the context of gluten sensitivity remains a fact.

## Data Availability

The datasets used and/or analysed for this project are available from the corresponding author on reasonable request.
